# Polycyclic Aromatic Hydrocarbons in Maternal and Umbilical Cord Blood from Pregnant Hispanic Women Living in Brownsville, Texas

**DOI:** 10.3390/ijerph8083365

**Published:** 2011-08-17

**Authors:** Ken Sexton, Jennifer J. Salinas, Thomas J. McDonald, Rose M. Z. Gowen, Rebecca P. Miller, Joseph B. McCormick, Susan P. Fisher-Hoch

**Affiliations:** 1 University of Texas School of Public Health, Brownville Regional Campus, 80 Fort Brown–AHC, Brownsville, TX 78520, USA; E-Mails: jennifer.j.salinas@uth.tmc.edu (J.J.S.); joseph.b.mccormick@uth.tmc.edu (J.B.M.); susan.p.fisher-hoch@uth.tmc.edu (S.P.F.-H.); 2 School of Rural Public Health, Texas A&M System Health Science Center, SRPH Building, College Station, TX 77843, USA; E-Mail: tmcdonald@srph.tamhsc.edu; 3 Texas Commission on Environmental Quality, Region 12, 5425 Polk Street, Houston, TX 77023, USA; E-Mail: rebecca.miller@tceq.texas.gov

**Keywords:** biomarkers, fetal exposure, maternal exposure, PAHs, prenatal exposure

## Abstract

Venous blood was drawn from 35 pregnant Hispanic women living in Brownsville, Texas, and matched cord blood was collected at birth. Gas chromatography/mass spectrometry was used to measure concentrations of 55 individual PAHs or groups of PAHs. Results indicate that these women and their fetuses were regularly exposed to multiple PAHs at comparatively low concentrations, with levels in cord blood generally exceeding levels in paired maternal blood. While the possibility of related adverse effects on the fetus is uncertain, these exposures in combination with socioeconomically-disadvantaged and environmentally-challenging living conditions raise legitimate public health concerns.

## 1. Introduction

There is convincing evidence that exposures to a variety of environmental chemicals can have adverse effects on children’s health [1–8]. The developing fetus and neonates are particularly at risk because they are known to be more susceptible than adults to the toxicological consequences of numerous environmental chemicals, including environmental tobacco smoke (ETS), lead (Pb), mercury (Hg), organochlorine and organophosphate pesticides, polychlorinated biphenyls (PCBs), and polycyclic aromatic hydrocarbons (PAHs) [1,2,7,9–11]. This increased sensitivity is attributable to, among other factors, higher rates of cell proliferation, decreased capability to activate and detoxify carcinogenic compounds, and lower immune-response capacity [7,9,12]. Prenatal exposure to PAHs is a particularly high-priority public health concern because these compounds are ubiquitous in various environmental media (e.g., air, water, soil, food), readily cross the placenta from mother to fetus, are known human carcinogens and suspected developmental and reproductive toxicants, and exposures have been documented for both pregnant women and their fetuses [13–18].

Chemicals classified as PAHs are made up of two or more fused benzene rings, and include hundreds of compounds [19], ranging in complexity from naphthalene (two rings), to phenanthrene and anthracene (three rings), to the more common five- or six-ring chemicals, like benzo(a)pyrene (five rings) and dibenz(a,h)anthracene (six rings). PAHs are produced as by-products of incomplete combustion of organic substances, and can also be found in crude oil, coal, coal tar pitch, creosote, roofing tar, and asphalt used for road construction. People are typically exposed to a complex mixture of PAHs from ingesting smoked, grilled, barbecued, or burned foods, eating contaminated uncooked foods (e.g., grown in contaminated soil), inhaling wood or tobacco smoke, breathing contaminated indoor or outdoor air (e.g., vehicle exhaust, incinerator emissions, fumes from asphalt roads and roofing tar), ingesting contaminated house dust and soil, drinking contaminated tap water (e.g., leaching from coal tar and asphalt linings in storage tanks and distribution lines), and skin contact with creosote-treated wood [20,21].

Although PAHs tend to be persistent in the environment, once inside the body they behave more like nonpersistent chemicals because they are metabolized rapidly, eliminated in the urine, and only small amounts are deposited in biological matrices [4]. Since the half-life of PAHs in blood is a matter of hours, concentrations are typically three orders of magnitude lower than urinary metabolite levels [4]. Consequently, most biomarker studies looking at PAH exposures have measured either PAH metabolites in urine [21] or DNA-PAH adducts in blood [13–18]. This article reports results from a novel study to measure actual concentrations of PAHs in blood from pregnant women and matched umbilical cords.

## 2. Methods

The investigation was designed to quantify PAH concentrations in whole blood from pregnant women and, subsequently, in a sample of cord blood taken at the time of birth. Participants in the study were volunteers recruited from the patient population at a private clinic in Brownsville, Texas (USA). Brownsville is located in south Texas along the U.S.-Mexico border, in a region known as the Lower Rio Grande Valley (LRGV). According to the U.S. Census Bureau [22], the city has a population of 172,434, of whom 92% are Hispanic. The 2006 American Community Service Survey ranked Brownsville as the most impoverished city in the nation based on average-annual household income [22]. More than a third of residents are 18 years old or younger, and 45% live in poverty; the highest proportion of any city in the U.S. with a population over 100,000 [22].

### 2.1. Subjects

Pregnant women in their first or second trimester presenting at a private gynecological clinic were told about the study and invited to participate. Informed verbal and written consent (either in Spanish or English, as appropriate) was obtained from those who agreed. No incentives were provided to participants, and the study received approval from the Committee for the Protection of Human Subjects at the University of Texas Health Science Center at Houston. Participants completed a short questionnaire on demographic and socioeconomic characteristics at the time of enrollment.

### 2.2. Specimen Collection and Handling

Between October 2005 and February 2006, venous blood samples were collected during routine clinic visits (third trimester) and cord blood was obtained at birth. The time between collection of maternal blood and collection of cord blood varied, with 6 matched maternal-cord sample pairs obtained within 24 hours of each other, 10 within 2–14 days, 16 within 15–35 days, and 3 within 43–57 days. Collection of maternal blood was accomplished by venipuncture, and samples were put into a 10 mL, red-topped, vacutainer tub, then labeled, and refrigerated. After the umbilical cord was severed at birth, approximately 10 mL of blood were drained into a red-topped plain vacutainer tube, which was capped, labeled, and refrigerated. For shipping, each unopened blood tube was sealed with Teflon tape and placed upright in an individual slot inside a pressure jar. Gel or ice packs were placed under, around, and over the jar, which was then sealed in a shipping container and shipped by overnight express to the laboratory.

### 2.3. Laboratory Analysis

All samples were analyzed in the laboratory at the School of Rural Public Health, Texas A&M University, in College Station, Texas [23]. Dichloromethane was added to the blood samples to start the extraction process once they arrived at the lab. Later, sodium sulfate was added and the mixture homogenized three times at 3 minutes per extraction. The combined extracts were filtered and concentrated to 3.0 mL. The samples were then cleaned on a silica/alumina column and subjected to high-performance liquid chromatography (HPLC). Afterward, samples were concentrated to the final volume of 0.5 mL. All blood samples were subsequently analyzed for more than 50 individual PAHs (e.g., benzo(a)pyrene) or groups of PAHs (e.g., C3-fluorenes) using a combination of gas chromatography and mass spectrometry (GC/MS) according to modified US Environmental Protection Agency SW-846 Method 8271C [24].

### 2.4. Not-Detected and Below-Detection-Limit Values

A significant fraction of blood samples usually fall below the limit of detection (LOD) in most exposure biomarker studies, which requires application of simple yet valid methods for reporting and statistically analyzing concentrations <LOD [25,26]. Numerous methods for dealing with left-censored (below LOD) data have been proposed [27,28], but there is no scientific consensus about the most appropriate one. The U.S. EPAg [29] and several scientists advocate using all instrument-generated values, including those <LOD, in order to have a complete and uncensored data set on which to perform statistical analyses [30–33]. The method LOD for all blood PAHs analyzed as part of this study was approximately 2 ng/mL. Compound-specific LODs and the number of measured blood PAH levels that fell into one of three categories (concentrations > LOD; concentrations > 0 but <LOD; and no instrument response *i.e.*, no analyte found by GC/MS) are summarized as part of Appendix A in Tables A1, A2, and A3. Statistical analyses used the instrument-generated values for the first two categories, and one-half (0.05 ng/mL) of the lowest reported concentration for the third category.

### 2.5. Data Analysis

The data were log normal, and geometric means and standard deviations were calculated for the 35 matched blood pairs and the six matched pairs collected within 24-hours of each other. Paired t-tests were used to evaluate whether observed differences between PAHs in cord blood and maternal blood were statistically significant. Pearson correlation coefficients were used to examine the strength of the statistical relationship between cord and maternal blood concentrations collected within 24 hours of each other.

## 3. Results

Demographic and socioeconomic characteristics of the 35 women enrolled in the study are summarized in Table 1. All the women were Hispanic, between the ages of 18 and 38 years old, and had lived in the Brownsville area for several years. Only one woman identified herself as an active smoker (however, her PAH concentrations were unremarkable). The mean height was 5.2 ft and mean weight was 161 lbs. The women had an average of 2.6 children at home and the average number of previous pregnancies was 2.8. Approximately 63% were born in the U.S., 6% in Mexico, and 31% elsewhere or unknown. Twenty percent graduated from high school or completed a GED, and an additional 49% graduated from a college or university. Seventy-one percent were married, 26% had never been married, and 3% were separated. Forty percent worked in an office, business, or shopping mall, 20% were housewives, and 20% were teachers, administrators, or students.

Cord and maternal blood were analyzed for 55 PAHs. As shown in Table 2, 19 compounds (34.5%) were not found at all and 10 compounds (18%) were only infrequently measurable. These 29 compounds were excluded from further statistical analysis but are included as part of the “total PAH” (sum of all measureable concentrations above zero) values reported in Tables 3 and 4.

Summary statistics for the other 26 PAHs and for total PAHs (sum of all concentrations above zero) are provided in Table 3. Geometric mean and geometric standard deviation for each compound are presented for cord blood, maternal blood, and the ratio of cord-to-maternal blood (C/M ratio). For all PAHs, geometric mean concentrations in cord blood are greater than or equal to those in maternal blood, and the C/M ratio is ≥ 1 except for acenaphthene. Based on two-tailed t-tests, observed differences between cord and maternal blood were statistically significant (p < 0.05) for 17 compounds and for total PAHs. The nine PAHs for which concentrations were not statistically different were naphthalene, C1-naphthalenes, biphenyl, acenaphthene, C3-fluorenes, 1-methylnaphthalene, 2-methylnaphthalene, 2,6-dimethylnaphthalene, and 1,6,7-trimethylnaphthalene.

Because most maternal-cord blood pairs (29 out of 35) were collected days to weeks apart, a direct comparison implicitly assumes that PAH exposures were relatively constant over the time period that data were collected. Although the data exhibit a consistent pattern, with cord PAHs tending to be greater than matched maternal values, information necessary to estimate PAH exposures is not available, but by focusing on the six matched pairs collected less than 24 hours apart (see Table 4), the relationship between PAHs in maternal and cord blood can be explored more directly (*i.e.*, the PAH exposure will be the same for both sample types).

As shown in Table 4, the general pattern is the same for the 6 matched sample pairs collected in close time proximity, with C/M ratios greater than 1.0 for all PAHs, except acenaphthene. The geometric mean C/M ratio for total PAHs is 2.3, compared to 1.9 for all 35 matched blood pairs. Relatively strong negative correlations (range −0.45 to −0.97) were observed between cord and maternal blood concentrations for 13 of 26 PAH compounds and for total PAHs.

## 4. Discussion and Conclusions

Pregnant women in the U.S. are regularly exposed to PAHs in the air they breathe, the water they drink, the food and beverages they consume, and the dust and soil they touch [26,34]. Environmental chemicals like PAHs can, depending on their intrinsic properties, cross the placenta by a variety of mechanisms such as facilitated diffusion, passive diffusion, active transport, and filtration. Once in the placenta, xenobiotic chemicals can be acted on by enzymes and metabolized into toxic or non-toxic metabolites that can enter the fetal blood stream, or they can accumulate [7].

Many PAHs have been shown to cross the placental boundary in experimental animal studies [35,36], and results indicate that fetal dose is at least an order of magnitude lower than maternal dose. Yet rodent bioassays also suggest that levels of fetal DNA-PAH adducts [37], DNA single-strand breaks and micronuclei formation [37,38] are higher compared to maternal tissues. Because PAH half-life in blood is relatively short, on the order of a few hours, human exposures are typically measured using either urinary metabolites [21] or DNA-PAH adducts in blood [13–18]. Although comparatively few direct measurements of blood-level PAHs have been reported, there is empirical evidence demonstrating that PAHs are present routinely in the human placenta [39] and that PAH concentrations in cord blood are often higher than in maternal blood [40–42]. Consistent with earlier studies, our results document generally higher PAH concentrations in cord compared to paired maternal blood for Hispanic women in Brownsville.

Although the significance of these findings for fetal health and development is unknown, there are reasons for concern. The National Toxicology Program (NTP) and the U.S. Environmental Protection Agency (EPA) list many PAHs as “known, probable, or possible” human carcinogens [43,44]. Animal bioassays have demonstrated that PAHs are transplacental carcinogens and that the fetus and neonate are more susceptible to PAH-caused carcinogenesis than adults [45,46]. Analogous data for humans are lacking, but it is clear that the human fetus is more vulnerable to the carcinogenic effects of pharmacological drugs like diethylstilbestrol [47]. In addition, PAHs have also been shown to adversely affect the immune system [48], reduce fetal growth [13], and cause harmful neurodevelopmental effects [18].

The 35 women enrolled in this study appear to be from a comparatively high socioeconomic stratum because, first, they sought out and could afford prenatal care at a private clinic and, second, as shown in Table 1, they were relatively well educated and most worked outside the home. Nevertheless, it is likely that they shared at least some of the common problems routinely encountered by the predominately Hispanic population of Brownsville, which exhibits many of the socioeconomic attributes known to be associated with poor health outcomes; including, poverty, illiteracy, inadequate housing, unemployment, use of English as a second language, substandard diet, lack of access to health care and, in general, more stressful and less healthful lives [49–51]. Residents often lack access to clean drinking water and adequate sewage treatment facilities, cannot afford air conditioning, experience frequent flooding, and are deficient in knowledge about healthy lifestyles [49,50]. Contaminated drinking and recreational water is a problem, air pollution is generated by motor vehicles, industrial facilities, agricultural operations, and open burning of trash, and the land is fouled by scrap yards, tire dumps, and pesticide use [52]. Brownsville residents experience above-average prevalence rates for many chronic diseases with an environmental component, including obesity, diabetes, tuberculosis, and cardiovascular disease [49–51], and children living along the Texas-Mexico border are hospitalized with asthma at a 36% higher rate than non-border children [46]. Overall, the available evidence suggests that pregnant women (and their fetuses) living in Brownsville are likely to experience elevated cumulative health risks from regular exposure to a complex concoction of chemical and nonchemical stressors [49–53]; a situation that could make them more susceptible to the adverse effects of PAHs and other environmental chemicals [54,55].

Because so few studies have reported direct measurements of PAHs in maternal and cord blood, and because those that have used different analytical techniques to monitor disparate populations, it is not possible to put measured PAH concentrations into perspective by comparing them to statewide or national distributions. However, we do know that the developing fetus is exquisitely sensitive to xenobiotic chemicals during certain time windows of vulnerability, and that even minute amounts of exogenous substances can produce adverse outcomes in the fetus or developing infant [3,4,7,8,10]. There is mounting evidence of reduced birth weight [15,17], decreased postnatal weight gain [16], smaller head circumference [14,15], impaired cognitive development [18], and increased susceptibility to procarcinogenic DNA damage [15] from prenatal exposure to relatively low levels of PAHs. This raises questions about the potentially harmful effects of relatively minimal PAH concentrations on the developing fetus, especially given its inherent biological vulnerability, the potential for synergistic interactions between multiple chemical and nonchemical stressors, and the likelihood that susceptibility is enhanced by socioeconomically-difficult and environmentally-demanding living conditions.

Our results demonstrate that pregnant Hispanic women and their fetuses residing in Brownsville are routinely exposed to a diversity of PAHs at comparatively low levels, and that concentrations in umbilical cord blood tend to be higher than in maternal blood. Although the cohort is a relatively-small convenience sample, and quantifiable PAH levels were often below the nominal limit of detection, nevertheless, results document *in utero* exposure to various PAHs, many of which are known neurodevelopmental toxicants and/or human carcinogens. Because women in our study were relatively better educated and more affluent than most residents of Brownsville, future studies should investigate exposures for the poorest and least educated segment of the Brownsville population, including an examination of possible associations between measured concentrations of exposure biomarkers and adverse health outcomes.

## Figures and Tables

**Table 1.** Sociodemographic attributes of women participating in the study (N = 35).

**VARIABLE**	**MEAN (S.D., Range)**
**Age (years)**	25.8 (5.5, 18–38)
**Height (feet)**	5.2 (0.21, 4.8–5.6)
**Weight (pounds)**	160.9 (36.9, 96–237)
**Previous Pregnancies**	2.8 (0.76, 2–4)
**Number of Children**	2.6 (0.5, 2–3)
**Country of Birth**	
United States	22 (62.9)
Mexico	2 (5.7)
Other or Unknown	11 (31.4)
**Education**	
Middle School	2 (5.7)
Some High School	8 (22.9)
Graduated High School/GED	7 (20.0)
Graduated College/University	17 (48.6)
Unknown	1 (2.9)
**Occupation**	
Housewife	7 (20.0)
Office/Business/Shopping Mall	14 (40.0)
Teacher/Student/Administrator	7 (20.0)
Outdoor Job	1 (2.9)
Unemployed	1 (2.9)
Other	4 (11.4)
Unknown	1 (2.9)
**Marital Status**	
Married	25 (71.4)
Never Married	9 (25.7)
Separated	1 (2.9)

**Table 2.** Summary of PAHs either not found or infrequently measurable in maternal and cord blood.

**No Concentration Measureable in 100% of Blood Samples**	
C1-benzothiphenes	C2-chrysenes
C2-benzothiphenes	C3-chrysenes
C3-benzothiophenes	C4-chrysenes
Naphthobenzothiphene	Benzo(k)fluoranthene
C1-naphthobenzothiophenes	Benzo(a)pyrene
C2-naphthobenzothiophenes	Indeno(1,2,3-c,d)pyrene
C3-naphthobenzothiophenes	Dibenzo(a,h)anthracene
C1-chrysenes	Benzo(g,h,i)perylene
C3-phenanthrenes/anthracenes	C3-dibenzothiphenes
C4-phenanthrenes/anthracenes	
**No Concentration Measureable in ≥ 80% to ≤ 99% of Blood Samples**	
Benzothiophene	C1-fluoranthenes/pyrenes
Acenaphthylene	C2-fluoranthenes/pyrenes
Carbazole	Benz(a)anthracene
Chrysene	Benzo(e)pyrene
Benzo(b)fluoranthene	Perylene

**Table 3.** Summary statistics for PAH concentrations in matched pairs of maternal and cord blood (N = 35).

**COMPOUND**	**CORD BLOOD GM (GSD) ng/mL**	**MATERNAL BLOOD GM (GSD) ng/mL**	**CORD/MATERNAL RATIO GM (GSD)**
Naphthalene	1.5 (1.6)	1.5 (1.3)	1.0 (1.7)
C1-Naphthalenes	1.1 (1.6)	1.0 (1.5)	1.2 (1.6)
C2-Naphthalenes	1.5 (1.7)	0.4 (7.8)	3.8 (7.3)
C3-Naphthalenes	0.8 (4.4)	0.2 (9.5)	4.6 (16.9)
C4-Naphthalenes	0.3 (10.7)	0.1 (11.9)	2.9 (11.8)
Biphenyl	0.5 (1.7)	0.5 (1.4)	1.1 (1.9)
Acenaphthene	0.0 (3.1)	0.0 (3.5)	0.6 (4.2)
Dibenzofuran	0.4 (1.7)	0.3 (2.9)	1.6 (3.5)
Fluorene	0.3 (1.9)	0.1 (4.1)	2.9 (4.9)
C1-Fluorenes	0.7 (2.0)	0.2 (6.5)	4.3 (6.8)
C2-Fluorenes	0.9 (3.0)	0.1 (8.6)	6.0 (9.8)
C3-Fluorenes	0.1 (8.8)	0.0 (5.0)	5.0 (23.1)
Anthracene	0.1 (2.9)	0.0 (3.1)	2.5 (4.3)
Phenanthrene	0.9 (1.8)	0.5 (2.4)	1.9 (2.6)
C1-P/A **^a^**	1.0 (2.0)	0.1 (7.3)	8.0 (7.1)
C2-P/A **^b^**	0.7 (2.8)	0.1 (7.3)	6.7 (9.6)
Dibenzothiophene	0.1 (2.5)	0.0 (3.5)	2.8 (5.2)
C1-DBT **^c^**	0.3 (2.5)	0.1 (4.8)	6.1 (5.9)
C2-DBT **^d^**	0.3 (3.7)	0.1 (5.0)	5.8 (6.4)
Fluoranthene	0.1 (2.1)	0.1 (3.3)	2.6 (4.0)
Pyrene	0.1 (2.6)	0.1 (3.3)	2.7 (4.9)
1-MNAP **^e^**	0.7 (0.7)	0.7 (1.6)	1.0 (2.1)
2-MNAP **^f^**	1.1 (0.7)	1.0 (2.0)	1.1 (3.4)
2,6-DMNAP **^g^**	0.5 (28.7)	0.2 (1.5)	2.7 (1.8)
1,6,7-TMNAP **^h^**	0.1 (8.4)	0.1 (1.1)	2.3 (1.1)
1-MPA **^i^**	0.2 (10.2)	0.1 (1.3)	3.4 (1.2)
Total PAHs **^j^**	14.1 (1.7)	7.4 (2.0)	1.9 (2.1)

a C1-phenanthrene/anthracenes;

b C2-phenanthrene/anthracenes;

c C1-dibenzothiophenes;

d C2-di-benzothiophenes;

e 1-methylnaphthalene;

f 2-methylnaphthalene;

g 2,6-dimethylnaphthalene;

h 1,6,7-trimethylnaphthalene;

i 1-methylphenathrene;

j Total PAHs = sum of all PAH concentrations >0 (including those not reported in the Table).

**Table 4.** Summary statistics for PAH concentrations in matched pairs of maternal and cord blood collected within 24-hours of each other (N = 6).

**COMPOUND**	**CORD BLOOD GM(GSD) ng/mL**	**MATERNAL BLOOD GM (GSD) ng/mL**	**CORD/MATERNAL RATIO GM (GSD)**
Naphthalene	1.5 (1.4)	1.2 (1.3)	1.2 (1.6)
C1-Naphthalenes	1.0 (1.3)	0.8 (1.3)	1.3 (1.5)
C2-Naphthalenes	1.3 (1.2)	0.5 (3.2)	2.4 (3.6)
C3-Naphthalenes	1.1 (1.5)	0.2 (3.8)	6.7 (5.3)
C4-Naphthalenes	0.9 (4.1)	0.2 (5.3)	3.7 (5.6)
Biphenyl	0.5 (1.4)	0.4 (1.4)	1.4 (1.9)
Acenaphthene	0.1 (1.3)	0.1 (1.5)	0.8 (1.8)
Dibenzofuran	0.4 (1.5)	0.3 (1.5)	1.2 (2.0)
Fluorene	0.3 (1.7)	0.1 (2.0)	2.5 (2.9)
C1-Fluorenes	0.7 (1.4)	0.1 (2.8)	5.1 (3.7)
C2-Fluorenes	0.9 (1.7)	0.1 (3.1)	6.4 (5.1)
C3-Fluorenes	0.3 (4.1)	0.1 (1.0)	5.5 (4.1)
Anthracene	0.1 (1.3)	0.1 (1.0)	1.8 (1.3)
Phenanthrene	0.7 (1.3)	0.4 (1.6)	1.8 (1.8)
C1-P/A **^a^**	0.8 (1.4)	0.1 (3.3)	5.5 (3,9)
C2-P/A **^b^**	0.6 (1.3)	0.1 (2.8)	4.5 (3.7)
Dibenzothiophene	0.1 (1.0)	0.1 (1.5)	1.4 (1.5)
C1-DBT**^c^**	0.3 (1.5)	0.1 (2.0)	3.5 (2.6)
C2-DBT**^d^**	0.3 (2.4)	0.1 (2.1)	2.6 (2.8)
Fluoranthene	0.1 (1.4)	0.1 (1.4)	1.6 (1.8)
Pyrene	0.1 (1.3)	0.1 (1.4)	1.8 (1.7)
1-MNAP**^e^**	0.7 (1.3)	0.5 (1.3)	1.3 (1.4)
2-MNAP**^f^**	1.1 (1.3)	0.8 (1.3)	1.4 (1.5)
2,6-DMNAP**^g^**	0.6 (1.4)	0.3 (2.2)	2.0 (2.7)
1,6,7-TMNAP**^h^**	0.1 (1.6)	0.1 (1.5)	1.5 (2.0)
1-MPA**^i^**	0.2 (1.6)	0.1 (1.5)	2.0 (1.9)
Total PAHs**^j^**	12.8 (1.3)	5.5 (1.7)	2.3 (2.1)

a C1-phenanthrene/anthracenes;

b C2-phenanthrene/anthracenes;

c C1-dibenzothiophenes;

d C2-di-benzothiophenes;

e 1-methylnaphthalene;

f 2-methylnaphthalene;

g 2,6-dimethylnaphthalene;

h 1,6,7-trimethylnaphthalene;

i 1-methylphenathrene;

j Total PAHs = sum of all PAH concentrations >0 (including those not reported in the Table).

## Appendix A

**Table A1.** Limits of Detection (LOD) for 19 Compounds that were not Measurable in 100% of Cord and Maternal Blood Specimens (N = 35 matched sample pairs).

**COMPOUND (PAH)**	**LOD ng/mL**
C1-Benzothiophenes	2.09
C2-Benzothiophenes	2.09
C3-Benzothiophenes	2.09
C3-Phenanthrenes/Anthracenes	1.74
C4-Phenathrenes/Antrhacenes	1.74
C3-Dibenzothiophenes	1.83
Naphthobenzothiophene	1.23
C1-Naphthobenzothiophenes	2.02
C2-Naphthobenzothiophenes	2.02
C3-Naphthobenzothiophenes	2.02
C1-Chrysenes	2.09
C2-Chrysenes	2.09
C3-Chrysenes	2.09
C4-Chrysenes	2.09
Benzo(k)fluoranthene	1.36
Benzo(a)pyrene	1.32
Indeno(1,2,3-c,d)pyrene	1.68
Dibenzo(a,h)anthracene	0.92
Benzo(g,h,i)perylene	0.87

**Table A2.** Limits of Detection (LOD) and Number of Cord and Maternal Blood Specimens (a) less than 0.1 ng/mL, (b) between 0.1 ng/mL and the Limit of Detection, and (c) greater than the Limit of Detection for 10 Compounds not Measurable in ≥80% to ≤99% of Samples (N = 35 matched sample pairs).

**COMPOUND (PAH)**	**LOD ng/mL**	**# of CORD SPECIMENS**			**# of MATERNAL SPECIMENS**		
		**<0.1^a^**	**0.1^a^–LOD**	**>LOD**	**<0.1^a^**	**0.1^a^–LOD**	**>LOD**
Benzothiophene	1.04	34	1	0	35	0	0
Acenaphthylene	1.16	34	1	0	33	2	0
Carbazole	1.96	28	7	0	29	6	0
C1-F/P^b^	2.02	30	5	0	33	2	0
C2-F/P^c^	2.02	35	0	0	34	1	0
Benzo(a)anthracene	0.77	35	0	0	30	5	0
Chrysene	1.04	35	0	0	32	3	0
Benzo(b)fluoranthene	1.74	35	0	0	34	1	0
Benzo(e)pyrene	1.83	35	0	0	34	1	0
Perylene	1.97	35	0	0	31	4	0

a ng/mL;

b C1-fluoranthenes/pyrenes;

c C2-fluoranthenes/pyrenes.

**Table A3.** Limits of Detection (LOD) and Number of Cord and Maternal Blood Specimens (a) less than 0.1 ng/mL, (b) between 0.1 ng/mL and the Limit of Detection, and (c) greater than the Limit of Detection for 26 Compounds included in the Statistical Analysis (N = 35 matched sample pairs).

**COMPOUND (PAH)**	**LOD ng/mL**	**# of CORD SPECIMENS**			**# of MATERNAL SPECIMENS**		
		**<0.1 ^a^**	**0.1 ^a^–LOD**	**>LOD**	**<0.1****^a^**	**0.1****^a^–LOD**	**>LOD**
Naphthalene	1.04	0	10	25	0	7	28
C1-Naphthalenes	1.99	0	24	11	0	26	9
C2-Naphthalenes	2.09	0	26	9	8	23	4
C3-Naphthalenes	2.09	3	24	8	13	18	4
C4-Naphthalenes	2.09	11	19	5	18	15	2
Biphenyl	0.87	0	34	1	0	35	0
Acenaphthene	0.77	25	10	0	18	17	0
Dibenzofuran	1.23	0	35	0	20	15	0
Fluorene	1.04	0	35	0	8	27	0
C1-Fluorenes	2.08	0	33	2	10	25	0
C2-Fluorenes	2.08	1	26	8	13	21	1
C3-Fluorenes	2.08	17	17	1	31	4	0
Anthracene	1.12	9	26	0	22	13	0
Phenanthrene	0.87	0	27	8	1	34	0
C1-P/A^b^	1.74	0	28	7	12	23	0
C2-P/A^c^	1.74	1	27	7	15	20	0
Dibenzothiophene	0.92	4	31	0	17	18	0
C1-DBT^d^	1.83	1	34	0	16	19	0
C2-DBT^e^	1.83	3	32	0	16	19	0
Fluoranthene	1.26	1	34	0	11	24	0
Pyrene	1.07	3	32	0	13	22	0
1-MNAP^f^	0.77	0	28	7	0	25	10
2-MNAP^g^	1.23	0	25	10	0	25	10
2,6-DMNAP^h^	1.23	0	35	0	8	27	0
1,6,7-TMNAP^i^	0.58	3	32	0	7	28	0
1-MPA^j^	1.23	0	35	0	6	29	0

a ng/mL;

b C1-phenanthrene/anthracenes;

c C2-phenanthrene/anthracenes;

d C1-dibenzothiophenes;

e C2-dibenzothiophenes;

f 1-methylnaphthalene;

g 2-methylnaphthalene;

h 2,6-dimethylnaphthalene;

i 1,6,7-trimethylnaphthalene;

j 1-methylphenanthrene.
